# Protein plasticity underlines activation and function of ATP-independent chaperones

**DOI:** 10.3389/fmolb.2015.00043

**Published:** 2015-07-28

**Authors:** Ohad Suss, Dana Reichmann

**Affiliations:** Department of Biological Chemistry, The Alexander Silberman Institute of Life Sciences, The Hebrew University of JerusalemJerusalem, Israel

**Keywords:** molecular chaperones, ATP-independent chaperones, intrinsically disordered proteins, protein homeostasis, protein conformation, stress repsonse

## Abstract

One of the key issues in biology is to understand how cells cope with protein unfolding caused by changes in their environment. Self-protection is the natural immediate response to any sudden threat and for cells the critical issue is to prevent aggregation of existing proteins. Cellular response to stress is therefore indistinguishably linked to molecular chaperones, which are the first line of defense and function to efficiently recognize misfolded proteins and prevent their aggregation. One of the major protein families that act as cellular guards includes a group of ATP-independent chaperones, which facilitate protein folding without the consumption of ATP. This review will present fascinating insights into the diversity of ATP-independent chaperones, and the variety of mechanisms by which structural plasticity is utilized in the fine-tuning of chaperone activity, as well as in crosstalk within the proteostasis network. Research into this intriguing class of chaperones has introduced new concepts of stress response to a changing cellular environment, and paved the way to uncover how this environment affects protein folding.

One of the key issues in biology is determining the mechanisms underlying the response of an organism to changes in the environment. The ability of cells to survive insults and recover after stress conditions is known to depend on a well-developed and complex network of protein chaperones and co-chaperones. In the late 70s, Roy Laskey introduced the term “molecular chaperones” to describe a role of DNA binding protein, nuceloplasmin, that facilitates the proper tertiary structure of nucelosome, and prevents aggregation of nucleosome particles in low ionic solution (Laskey et al., 1978). This definition was later on extended to protein molecular chaperone following elegant studies from the Hugh. Pelham group on Hsp70-BiP interactions in cells (Munro and Pelham, 1986). Twenty years later, a wealth of knowledge has been amassed regarding the activation, mechanism, and conservation of molecular chaperones. It is clear that these proteins, and the crosstalk between them, are essential factors in the maintenance of a “healthy” proteome in cells during normal and stress conditions (Bukau et al., 2006). Chaperones do not act in an independent fashion, rather they form complex and dynamic networks of chaperones and co-chaperones. This network is highly dynamic, and its composition readily adapts to environmental and endogenous changes (Jakob et al., 1999; Bukau et al., 2006; Hinault et al., 2006; Morimoto, 2008; Hartl et al., 2011; Bardwell and Jakob, 2012; Taipale et al., 2014). Protein folding is an intricate, multistep mechanism, involving binding of the nascent chain to a chaperone, followed by refolding and rebinding to the same or different members of the proteostasis network (Bukau et al., 2000; Preissler and Deuerling, 2012; Brehme et al., 2014; Cho et al., 2015). Directionality of the protein folding process is thought to be promoted by differences in substrate recognition between the various types of molecular chaperones (e.g., DnaK and GroEL). In bacteria, the newly synthesized, misfolded intermediates are first recognized by DnaK (Hsp70 in eukaryotes), followed by a cascade of binding-refolding events. In the event of unsuccessful refolding, the misfolded client protein is transferred to another chaperone complex, the chaperonin GroEL (Hsp60 and Hsp20 in eukaryotes), which has a wider promiscuity for partially folded substrates than DnaK/J (Buchberger et al., 1996; Houry, 2001). Thus, differences in structural (or/and sequence) properties of client proteins define the sequence of folding events, and the type of folding system. This cross-talk is one of the most essential and fascinating features of cellular chaperones and is mediated by a wide repertoire of co-chaperones. On the other hand, a sequential mode of action has accompanying drawbacks, particularly during stress conditions when a rapid response to an increased concentration of misfolded proteins is essential. Thus, the recruitment of additional chaperones and co-chaperones, specifically activated during stress conditions is extremely valuable.

Molecular chaperones can be classified based on their size, cellular localization, mode of action, substrate specificity, and other features. Here we propose to analyze two different types of classifications, based on mode of action and energy dependence. Following this classification, molecular chaperones can be divided to three main classes, “foldases,” assisting in protein refolding, “holdases” (or “holding” chaperones, Sharma et al., 2009; Priya et al., 2013) preventing protein aggregation by forming very stable complexes with misfolded intermediates, and “translocases,” escorting proteins to the correct cellular location, and maintaining their client proteins in a largely unfolded state until their successful incorporation into a membrane. A further type of classification is based on the energy dependence of chaperones: ATP-dependent and ATP-independent chaperones. ATP-dependent chaperones, usually “foldases,” (e.g., DnaK, GroEL, Hsp60, Hsp70, Hsp90) utilize cycles of ATP hydrolysis coupled with massive conformational changes to recognize, refold and release their client proteins. Mechanisms underlying the structural changes, oligomerization, and dynamics of ATP-dependent chaperones were extensively reviewed in Mayer (2010), Hartl et al. (2011), and Li et al. (2012).

In this review we choose to focus on an extremely diverse class of molecular chaperones, with one common feature—the ability to prevent protein aggregation with minimal energetic cost. We united all these chaperones in one class, termed ATP-independent chaperones.

The ATP-independent chaperones prevent protein aggregation in an energy-independent fashion, usually acting as “holdases,” rescuing the misoflded proteins without supporting a subsequent refolding of the substrate. In addition to the chaperone activity, some of the ATP-independent chaperones have additional catalytic activity, for example, as an isomerase (Schmidpeter and Schmid, 2015) or reductase (Kern et al., 2003; Rand and Grant, 2006; Teixeira et al., 2015).

Several members of this class of chaperones were discovered in prokaryotes and eukaryotes. The majority of these ATP-independent chaperones efficiently prevent protein aggregation induced by a particular stress (e.g., heat, oxidative unfolding, acidification, dryness) and form highly stable complexes with their client proteins (usually in nM range affinities), “holding” misfolded proteins from aggregation during stress conditions. Substrate release is then mediated by post-translational modifications which induce conformational changes in the chaperone and may be coupled (or not) to a related ATP-dependent foldase system for full reconstruction of the protein (Bardwell and Jakob, 2012). For many of the ATP-independent chaperones however, the release mechanism is not fully understood, and neither is their role in targeting irreversibly misfolded proteins for proteolysis.

ATP-independent chaperones are quite diverse in their stress response, structure and mode of action, and subclassifications of this group may be based on their stress specificity, architecture, ability to refold bound client proteins, as well as their crosstalk with other foldase and holdase chaperones.

Probably they have evolved independently in prokaryotes and eukaryotes, depending on the type of stress, and mode of activation. Even more strikingly, it was suggested that small heat shock proteins, sHSP, have evolved independently within their own sub-family (Haslbeck and Vierling, 2015). An intriguing issue is the reason for the evolution of multiple types of chaperones to deal with unfolding conditions and the question as to why efficient and highly abundant ATP-dependent chaperones should require the assistance of energy-independent chaperones? One possible reason is that under conditions of ATP depletion induced by stress, for example, oxidative (Winter et al., 2008) and acidic stress (Sun et al., 2011), cells must rely on ATP-independent chaperones. During oxidative stress, redox-regulated chaperones, Hsp33 (Jakob et al., 1999; Winter et al., 2008), RidA (Muller et al., 2014), Cys-2-Peroxiredoxin (Banerjee et al., 2015), Get3 (Powis et al., 2013; Voth et al., 2014) are rapidly activated by oxidation of specific “sensing” cysteine residues, that lead to productive chaperone activity and inhibition of massive protein unfolding in cell. Another possible reason for the evolution of ATP-independent chaperones is their ability to function in cellular compartments devoid or limited in ATP, such as the periplasm in bacteria, and the extracellular matrix in multicellular eukaryotes. Recently, numerous periplasmic ATP-independent chaperones have been characterized (Goemans et al., 2014), including: acid-regulated chaperones HdeA and HdeB (Malki et al., 2008; Dahl et al., 2015), Skp (Walton et al., 2009), and Spy (Quan et al., 2011). In mammals, the extracellular matrix protein, Clusterin, was implicated as a key ATP-independent player in intra and extra-cellular proteostasis (Poon et al., 2000; Wilson and Easterbrook-Smith, 2000; Narayan et al., 2012). Clusterin was found to be colocalized with senile amyloid plaques, and efficiently sequestered oligomeric forms of amyloid peptides (Narayan et al., 2012) as well as other misfolded proteins (Poon et al., 2000; Wilson and Easterbrook-Smith, 2000). However, ATP depletion is definitely not the main driving force for shaping diversity among ATP-independent chaperones. Therefore, additional explanations to explain the diversity and importance of ATP-independent chaperones in maintaining proteostasis in the cytosol, ER, and mitochondria should be considered. One possibility is that the mode of substrate recruitment and stress-regulated “holding” activity of these chaperones is mechanistically different from that of ATP-dependent chaperones, broadening the scope and enhancing the efficiency of defense system against the damage that misfolded proteins present for the cell.

## Structural dynamics of ATP-independent chaperones underlines their activity

One common feature of most ATP-independent chaperones is a regulated protein plasticity which is crucial to their activation, function and crosstalk with associated foldases.

In this review, we will focus on the types and roles of the structural dynamics in ATP-independent chaperones, required for efficient maintenance of protein homeostasis in cells during conditions that favor unfolding.

**Figure 1:**
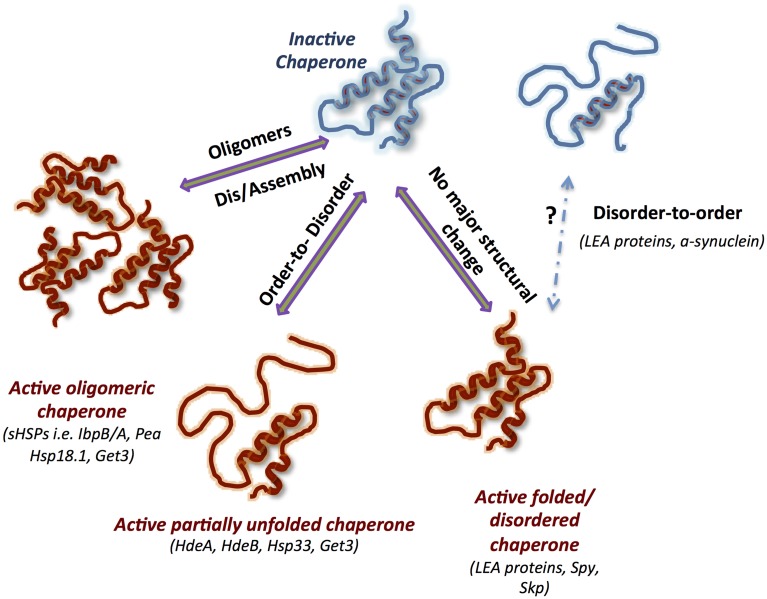
**Structural dynamics of ATP-Independent chaperones underlines their function**. Schematic representation of the main modes of actions mediated by conformational changes: (1) oligomerization, (2) order-to-disorder, and (3) disorder to-order transformations, as well as a mode of action used by constitutively unfolded or folded chaperones that does not require massive conformational changes. The inactive chaperones are blue, and the active chaperones are red. Known chaperones utilizing one or more of the specific mode of action are listed in brackets.

The modes of action of ATP-independent chaperones are as diverse and unique as the chaperones themselves (Figure 1). The majority of known ATP-independent chaperones require major conformational changes or/and oligomeric rearrangements for either activation or inactivation. Oligomerization has been shown to be crucial for the activity of some chaperones, while others require disassembly of oligomers to function. In addition, the active folded state of the chaperone, the tertiary structure, may or may not change during the course of its action. Undoubtedly, dynamics is a key note of the chaperone activity of most ATP-independent chaperones and, in consequence, is highly specific and regulated.

Nevertheless, there is yet poorly understood class of ATP-independent chaperones that do not change their tertiary structure during activity. Interestingly, as will be described later, introducing flexibility into the structure of one such chaperone, Spy, resulted in enhancement of the chaperone activity.

## Oligomerization and its role in chaperone activity of ATP-independent chaperones

**Figure 2:**
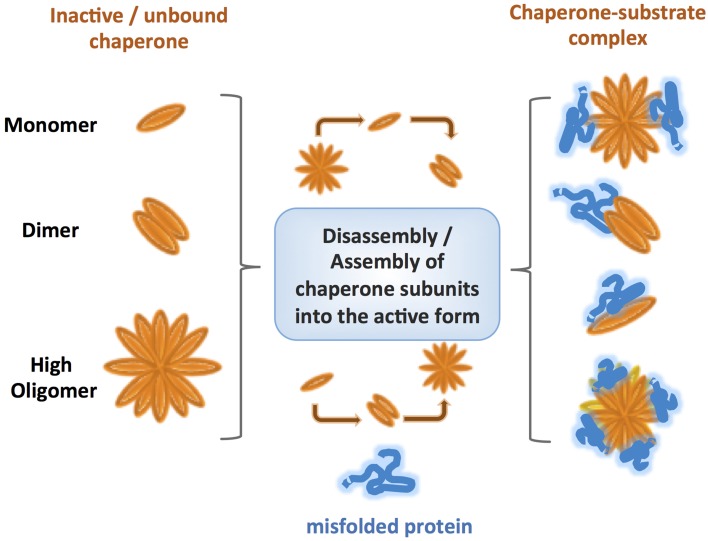
**Comparison of various types of oligomerization observed for different ATP-independent chaperones**. Under normal conditions the inactive chaperones are present either as monomers, or oligomers. However, under specific stress conditions, post-translational modifications lead to reorganization of the chaperone to either lower or higher state oligomers, promoting binding of the substrate without input of energy. The oligomeric states usually form highly dynamic ensembles with different conformations, which are thought to be crucial for a rapid response and fruitful binding of misfolded client proteins. Even within the same class, different chaperones follow diverse mechanisms of oligomer assembly and disassembly.

One of the most striking features of ATP-independent chaperones is their ability to assemble into oligomeric ensembles (Giese and Vierling, 2002; Lee et al., 2009; Haslbeck and Vierling, 2015; Teixeira et al., 2015). Wide differences in the oligomeric architecture and its role in chaperone activity were described for different families of chaperones, and for chaperones within the same family (Figure 2). Nevertheless, it is clear that these oligomeric structures: (1) are crucial for chaperone activity and substrate release, and (2) are highly dynamic. One of the proposed reasons for the formation of highly oligomeric structures is the increase in the local concentration of active chaperones during stress, as a necessary step in mounting a rapid defense. However, this cannot explain the observations that in some cases the active chaperone has a lower oligomeric state than the inactive form, requiring further consideration to define the role of oligomerization in maintaining proteostasis. To date, the roles of the best characterized oligomeric architectures were described for the small heat shock (sHSP) chaperones (Haslbeck and Vierling, 2015). The conserved αB-crystallin domain of sHSPs is known to govern the formation of dimers while the flexible N and C-termini mediate oligomeric interactions and substrate binding (Jaya et al., 2009; Rajagopal et al., 2015). Determination of the precise oligomeric architecture and its dynamics throughout the activity cycle of the chaperones is challenging because of the transient nature of the interactions and fast subunit exchange. Pea Hsp18.1, for example, can exist in 300 different chaperone-substrate stoichiometries (Stengel et al., 2010). With increasing temperatures, the Pea Hsp18.1 chaperone shifts from an arrangement of monodisperse dodecameric oligomers to higher-order oligomers varying in subunit number. This transition is initiated by disassembly of the dodecamers to suboligomeric species which are then reassembled to produce the active form of the chaperone. Such a dissociation-association mechanism is also shared by the bacterial chaperone IbpB (Jiao et al., 2008) and Hsp16.3 of *Mycobacterium tuberculosis* (Giese and Vierling, 2002; Gu et al., 2002; Fu et al., 2003; Fu and Chang, 2004). Mutations in the oligomerization domain of *Synechocystis* Hsp16.6 resulted in decreased chaperone activity and lowered the yield of refolded substrate while increasing the oligomeric stability (Giese and Vierling, 2002). The requirement for oligomers to have short-term binding and transient interactions, therefore, might be important for the transfer of unfolded substrates to ATP-dependent chaperones for refolding.

Additional complexity can be found in the heteromeric interactions between different sHSPs, such as the αA and αB hetero-oligomer in the lens (Stengel et al., 2010; Delbecq and Klevit, 2013). It is not yet clear how common hetero-oligomer formation is among ATP-independent chaperones or how important this phenomenon is for their function. However, it poses interesting questions regarding the physiological diversity of functional chaperone oligomers *in vivo* and its role in substrate recognition and targeting for refolding or degradation of the substrate in cells.

While the majority of studied oligomeric structures of ATP-independent chaperones are highly dynamic, examples of static oligomers do exist. Yeast Hsp26, was shown by Franzmann and colleagues to become active by a conformational change while maintaining the same oligomeric structure (Franzmann et al., 2008). It is safe to assume that this is not an isolated example.

Recent studies revealed interesting examples of oligomerization-driven activity in other, non sHSP chaperones, including Get3 and peroxiredoxin. In the reduced form, the Get3 protein is a dimeric ATPase, involved in binding and targeting tail-anchored proteins (TA) to the endoplasmic reticulum membrane. Upon oxidation of redox-sensitive cysteines, the Get3 protein loses ATPase activity and is reorganized into tetrameric and higher oligomeric structures with ATP-independent chaperone activity. The oligomeric assembly is fully reversible, mediated by the redox status of Get3 (Voth et al., 2014).

Overall, dynamic oligomerization and the ability to switch between oligomeric states appear to be crucial for the function of a variety of known chaperones, but in very different ways. The great diversity of ATP-independent chaperones with respect to oligomerization reflects the flexibility and unique function of each subgroup. Different rates of subunit exchange and sizes of oligomers might affect chaperone activation, inactivation, substrate specificity and release. One option is that oligomerization serves as a “rescue center,” assisting the misfolded protein to find shelter, and facilitating detection of the client substrate by the related foldase chaperones. Despite intense investigation, there remain many open questions that have to be addressed, for example: is there a limit for the number of subunits in the oligomers? Do allosteric effects drive oligomerization and does substrate binding effect this allosteric regulation? How do the oligomeric state and its dynamics operate *in vivo*? Is there a correlation between the biophysical properties of substrates and the oligomeric state of the ATP-independent chaperone? Due to the extremely fast diffusion rate of proteins in cells (Phillip et al., 2012), does the “local concentration” theory hold?

## Order-to-disorder transitions in ATP-independent chaperones, and their role in chaperone activity

### Protein disorder – biophysically poorly defined term

Over the last decade many examples of proteins harboring intrinsically disordered regions, have been identified and characterized (Flock et al., 2014; Fuxreiter et al., 2014; Liu and Huang, 2014; Oldfield and Dunker, 2014; van der Lee et al., 2014). It is important to note that protein disorder, and particularly “intrinsic disorder,” are not very precise terms. One of the most common definitions of an intrinsically disordered protein is the lack of a stable secondary structure under physiological conditions (Babu et al., 2011; van der Lee et al., 2014). It has been suggested that protein conformation space is highly heterogeneous, ranging from fully structured proteins with low flexibility at the loop regions to fully unstructured areas, resembling random coil structures. This conformational spectrum covers multiple states including variable sizes of flexible regions, molten globules and extended secondary structural elements. Based on this type of model there are no boundaries between different states, and therefore it is very challenging to define or map such structures precisely. A wide repertoire of experimental methods, including NMR, SAXS, FRET, and structural mass spectrometry combined with computational approaches have been used in recent years to shed light on the dynamic and physical properties of intrinsically disordered proteins. However, even with the great advance in methods of structural resolution, there is still progress yet to be made.

As a further complication, underlying all studies in this field is a lack of clear definition and differentiation between such terms as “unfolded,” “folded,” “partially folded,” “ordered,” and “disordered” which can be interpreted in a variety of ways. In this review we use the descriptive terms for protein state in their most general form, to indicate the existence or absence of a defined secondary structure.

### Intrinsically disordered chaperones

The majority of identified intrinsically disordered proteins were proposed to be involved in cellular signaling, or as scaffold proteins (van der Lee et al., 2014; Wright and Dyson, 2015). A more poorly characterized but expanding group of intrinsically disordered proteins includes molecular chaperones that use their structural plasticity to fulfill their function (Bardwell and Jakob, 2012; Kovacs and Tompa, 2012; van der Lee et al., 2014).

Due to experimental and conceptual challenges in studying intrinsically disordered proteins (e.g., lack of high resolution methods to map conformational changes, biochemical challenges in working with unfolded proteins) and due to large variability of already characterized mechanisms that different intrinsically disordered chaperones adopt, it is difficult to precisely define a general molecular mechanism of action of this class of chaperones. As it seems today, there is a broad scope of possible mechanisms that intrinsically disordered chaperones can adopt, based on degree of their unfoldeness, and their physiological role.

**Figure 3:**
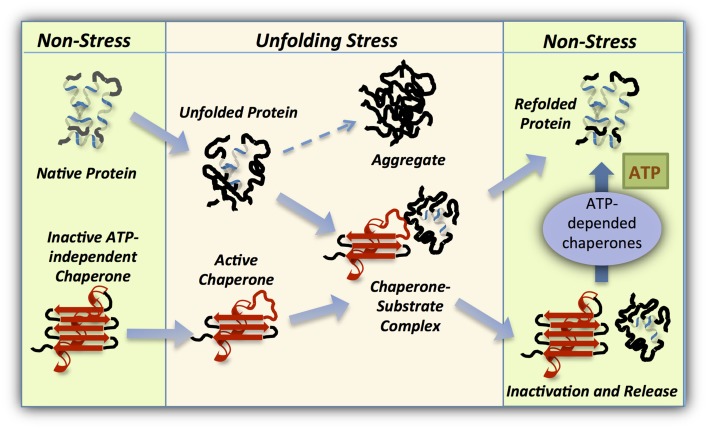
**Working cycle of intrinsically disordered chaperones**. Under normal conditions members of this class of ATP-independent chaperones (red) are folded and inactive. However, cellular conditions leading to unfolding of cellular proteins activate the holdase chaperone as a result of the same unfolding. This stress-regulated conformational changes in the chaperone exposes the substrate binding domains, enabling tight binding with the client protein. On return to non-stress conditions, the holdase chaperone is stabilized and refolded to release the substrate which may then be refolded in an ATP-dependent manner by an associated foldase chaperone.

One such mechanism, utilizes a stress regulated structural order-to-disorder transition to activate chaperone function, leading to the formation of stable complexes between the partially unfolded chaperone and misfolded client protein. A return to non-stress conditions permits the reverse disorder-to-order transition, enabling substrate release or transfer to a foldase chaperone (Figure 3). It is important to note that the definition of protein disorder is not strictly accurate for this type of proteins, since the folded structure is present in the inactive form and therefore the term: “conditional disorder” was introduced to describe this mode of action (Bardwell and Jakob, 2012).

A notable example of a conditionally disordered chaperone is the acid-regulated HdeA, which is activated in response to acidic stress in the ATP-depleted periplasm of bacteria (Malki et al., 2008; Tapley et al., 2009). Under neutral conditions, HdeA exists as an unstable dimer but with decreasing pH, charged residues on the dimer interface become protonated leading to monomerization and unfolding of the HdeA monomers. These unfolded or partially unfolded monomers bind a large range of substrates, which have themselves unfolded in the acidic conditions (Zhang et al., 2011). Recent studies concerning HdeB a closely related homolog of HdeA, showed that the chaperone activity of HdeB is optimal at slightly higher pH values than for HdeA (Kern et al., 2007; Dahl et al., 2015). It was suggested that bacteria, particularly intestinal bacteria, such as *Escherichia coli*, need both HdeA and HdeB in order to span different ranges of physiologically relevant acidity, and survive in the mammalian gut (Dahl et al., 2015).

The bacterial (Ilbert et al., 2007; Reichmann et al., 2012) and algae (Segal and Shapira, 2015) chaperone Hsp33 operates a similar mechanism to that of HdeA. Under oxidative stress, this redox-regulated chaperone undergoes significant unfolding and reveals a flexible redox-sensing domain, which triggers its chaperone activity (Ilbert et al., 2007; Reichmann et al., 2012). This unfolding process is the result of the formation of disulfide bonds and the release of a bound zinc ion which stabilize this domain in the reduced, inactive, form of Hsp33. Reduction of the Hsp33-substrate complex results in a refolding of Hsp33, which generates enough energy to release the bound misfolded substrate to the DnaK/J system for correct refolding (Hoffmann et al., 2004).

A transition from a well-folded to unfolded state has also been demonstrated in small heat shock proteins, such as yeast Hsp26 which responds to heat shock by unstabilizing its middle thermosensing domain (Franzmann et al., 2008). Intrinsically disordered fragments may enhance the promiscuity of substrate binding in a unique conformation for each partner, and promote the formation of affinity complexes in the nM range (Flock et al., 2014; Liu and Huang, 2014). Such enhancement may be achieved by exposure to stress conditions, as in the case of IbpB which becomes highly active and greatly flexible after heat shock stress (Jiao et al., 2008).

Once stress conditions are relieved, activated chaperones can return to their inactive state, accompanied by substrate release and refolding. The rate of chaperone refolding might have significance for the refolding of the substrate itself, or/and the recruitment of another member of the proteostasis network to complete the job. Rapid refolding of cytosolic Hsp33 leads to the transfer of its substrate to the foldase system (Hoffmann et al., 2004; Reichmann et al., 2012) while the perplasmic HdeA refolds at a much slower rate, enabling a slow release of the substrate into the periplasmic matrix (Tapley et al., 2010). This mechanism maintains low concentrations of unfolded proteins in the ATP-depleted environment of the periplasm, thus preventing post-stress aggregation and permitting gradual refolding or proteolysis of the substrate.

In summary, conditionally disordered chaperones employ order to disorder transitions as a method of activation, and disorder to order changes as a means of releasing their substrate. This unique feature makes these chaperones well-suited to respond rapidly to protein unfolding following stress.

## Constitutively unfolded ATP-independent chaperones

The LEA proteins are a well described class of entirely disordered proteins which are key players in cell survival during abiotic stress in plants. LEA proteins are overexpressed in plants during seed formation, as well as in vegetative tissues during drought (Mouillon et al., 2006; Kovacs et al., 2008a,b; Popova et al., 2011; Hincha and Thalhammer, 2012). Although they were initially identified in plants, homologs of LEA proteins have been identified in bacteria, invertebrates and also in vertebrates living in extreme environments. Being very diverse in their structure and functions, their role as protein shields during abiotic stresses such as dehydration or exposure to low temperatures, makes LEA proteins intriguing and extremely challenging research subjects.

The chaperone abilities displayed by some of the LEA proteins are incredibly varied, ranging from low holdase activity to activities comparable to highly efficient molecular chaperones. Several LEA proteins have been shown to adopt an ordered conformation, mostly α-helical structure, under stress conditions, raising a question concerning their structure in the active state. An increase in crowding conditions did not promote their folding suggesting that, most probably LEA proteins are active in their unfolded or partially unfolded state. Moreover, recent evidence showed that apart from their activity as holding chaperones, fully disordered LEA chaperones play a role in membrane stabilization in multiple stress conditions. It is possible that interaction with lipids might affect the folding and activity of members of this chaperone class.

Other examples of intrinsically disordered proteins with holding activity include α-synuclein which has been extensively studied due to its association with Parkinson's disease, and a milk protein, α-casein (Kovacs and Tompa, 2012). These proteins possess lower levels of chaperone activity than other molecular chaperones, suggesting a different mechanism for their holding activity. It was suggested that α-synuclein interacts with phospholipids that affect its chaperone activity. For casein, the physiological meaning of the chaperone activity is not clear. Although the chaperone activity may be incidental, casein can be used as a model for studying the chaperone activity of an intrinsically disordered protein. A final outstanding example of a constitutively unfolded chaperone is *S. cerevisiae* Hsp12, which lacks the conserved α-crystallin domain characteristic of most small heat shock proteins, and is fully disordered (Welker et al., 2010; Kovacs and Tompa, 2012). Upon extreme heat stress this molecular chaperone translocates from the cytoplasm to the cell membrane, increasing membrane stability and promoting cell survival.

The mechanism by which fully disordered chaperones execute their activity is poorly understood. The abundance of hydrophilic and charged residues in the sequence of the LEA chaperones, may allow them to serve as hydration buffers, retaining water during dehydration. Besides their hydrophilic nature, it is possible that the constitutive disordered conformation allows for flexible substrate recognition by these chaperones, similarly to that achieved by the conditional disorder induced by stress in other chaperones. It is as yet unknown whether chaperones like the LEA proteins are promiscuous or specific in their binding, and whether they favor structurally disordered clients over correctly folded ones. Their ability to adopt a folded (or partially folded) conformation under stress conditions might play a role in their binding mechanism, helping to stabilize chaperone and substrate together as a soluble complex while preventing entropic collisions with unfolded substrates.

Due to difficulties in obtaining structural information related to the unfolded chaperones and their aggregation-prone substrates, especially in physiological conditions, the degree and process of unfolding during the chaperone activity cycle is not fully characterized. Future research on this type of chaperones under unfolding conditions, will be necessary to shed a light on the role of protein plasticity in cell defense.

## Well-folded ATP-independent chaperones

Intrinsic disorder has been defined as a staple feature of chaperone activity, allowing them to recognize a wide range of substrates and preventing their aggregation. This principle was demonstrated above by conditionally and constitutively disordered types of known chaperones. Nevertheless, recent studies have discovered an additional class of ATP-independent chaperones that are characterized by a rather structured conformation with no significant or detectable disorder. Among these is the newly discovered periplasmic chaperone, Spy, which is folded in a cradle shaped conformation with short and flexible N and C-termini (Quan et al., 2011, 2014). Spy binds its substrates mostly through hydrophobic interactions conferred by the hydrophobic patches on its surface. Although Spy is generally less unstructured than most studied chaperones, mutagenesis leading to a greater degree of flexibility has been shown to increase the binding ability (Quan et al., 2014). Notably, this degree of disorder must be limited by the point at which the binding becomes too strong to allow the eventual release of the unfolded substrate.

Similarly to Spy, the bacterial periplasmic chaperone Skp has no evident intrinsic disorder but still possesses chaperone activity (Walton and Sousa, 2004). The crystal structure of Skp revealed a trimeric protein resembling a jellyfish with α-helical arms protruding from a β-barrel central cavity. Unfolded substrates are bound and isolated from the solution inside this central cavity, while the α-helical arms permit the chaperone great flexibility. The same attributes are found in a very similar eukaryotic cytosolic chaperone, prefoldin, which assists in binding cytoskeletal unfolded proteins. Not very surprisingly, Spy itself resembles Skp with a jellyfish-like α-helical bundle.

## Substrate specificity of ATP-independent chaperones

ATP-independent chaperones are involved in the promiscuous binding of many different substrates. How these chaperones are able to distinguish between the entire cell proteome and preferentially bind certain substrates over others, remains an open question. Many studies have focused on the characterization of the binding sites of different chaperones, while others attempted to determine the common structural and sequence features of the substrates (Alcock et al., 2008; Fu et al., 2014).

In general, chaperones bind aggregation-prone proteins by recognizing their internal hydrophobic segments, which have become exposed due to unfolding or poor refolding. Studies have demonstrated that sHSPs bind fully disordered clients more efficiently than partially unfolded ones (Hoffmann et al., 2010; Kovacs and Tompa, 2012). However, redox-regulated chaperone, Hsp33, has a strong preference for partially folded substrates which unfold further after the inactivation of Hsp33 and become appropriate substrates for transfer to the DnaK/J system (Reichmann et al., 2012). These results hint at the existence of a selective mechanism, not yet fully characterized, by which ATP-independent chaperones recognize their substrates (Fu et al., 2014).

Most studies of chaperone activity have used model substrates, such as firefly luciferase, porcine citrate synthase and malate dehydrogenase which, while successful, are not natural *in-vivo* substrates of most of the studied chaperones. Recently, the natural substrates of bacterial sHSPs, specifically *E. coli* IbpB and *D. radiodurans* Hsp20.2 chaperones have been investigated (Fu et al., 2013). The two sHSPs differ from each other with respect to their oligomeric state and substrate range. About 100 different substrates of IbpB and Hsp20.2 were identified, by analyzing chaperone-substrate complexes *in-vivo* and a preference for translation-related proteins and certain metabolic enzymes was detected. Fu et al. compared the two substrate collections of IbpB and Hsp20.2 using bioinformatics. The results suggested that these two chaperones tend to bind proteins of high molecular weight and with abundant charged residues (Fu et al., 2013).

Due to their flexible nature and sometimes disordered conformation, chaperones can interact with many sets of substrates differing in molecular weight, charge, hydrophobicity and structure and this complicates a characterization of substrate specificity. The interactions also involve different domains within the chaperone which may stabilize the client proteins in distinctive ways. IbpB and PsHsp18.1 has been shown to interact with proteins mostly through the N-terminal domain (Jaya et al., 2009; Fu et al., 2013), although in many cases the α-crystallin domain is also involved in substrate binding (Ganea, 2001; Fu et al., 2013). The ability of ATP-independent chaperones to utilize their entire sequence and structure to interact specifically with any substrate must be taken into account when studying their natural substrates and binding sites.

## The cross talk between different types of chaperones

The ultimate goal of the disaggregation function of holdase chaperones, besides preventing the destructive outcome of the aggregation itself, is to create a large reservoir of misfolded proteins that can be refolded back into their native structure or be targeted for proteolysis. This is extremely useful after cells experience stress leaving the transcription and translation machineries damaged or lacking the requisite ATP for function. Once the stress is relieved, the holdases conditionally transfer the bound pool of substrates onto the cellular folding or degradation systems, fulfilling a vital function in the recovery of the cell (Bardwell and Jakob, 2012; Haslbeck and Vierling, 2015).

The different families of molecular chaperones act together as a network in which each family member has its distinct features and specific function. As a part of that network, holdase chaperones are able to communicate with other chaperones in the network, to achieve refolding of misfolded clients (Figure 3). Crosstalk between chaperones has been shown to exist in organisms of all classes, prokaryotes and eukaryotes alike. Yet, a crasstalk between different types of ATP-independent chaperones was not well characterized.

A catalytic triad comprising sHSPs, ClpB, and the DnaK folding system has been described (Mogk et al., 2003). The role of the sHSPs in this triad is to form an insoluble complex with substrate proteins during stress. These complexes can later undergo disaggregation by ClpB and subsequent refolding by the DnaK/J/E system. All three partners communicate with each other through mechanisms that are still unclear. Similarly, in the case of Hsp33 and DnaK/J system, the exact transfer mechanism is not fully understood.

As described previously, a number of ATP-independent chaperones are able to promote protein refolding independently of ATP-dependent foldases, especially in ATP depleted environments. In the periplasm for example, a lack of ATP renders ATP-dependent chaperones useless, leaving chaperones such as Spy, Skp, and HdeA responsible for reconstituting the periplasm proteome after stress is relieved. However, there is a possibility that also these chaperones are able to transfer their client proteins to another chaperones, enhancing the substrate refolding or degradation.

What makes a certain chaperone obligated to a foldase or else foldase-independent is unclear. While foldases clearly supply substrates with the energy and environment needed for quick refolding, chaperones in ATP-depleted sections of cells are able to bring about the same outcome by an unknown mechanism. While chaperone structure and binding domains play a significant role in this process, the release kinetics are crucial to the outcome.

## Outlook

Research over the past decade has revealed fascinating insights into the diversity of ATP-independent chaperones, and the variety of mechanisms by which structural plasticity is utilized in fine-tuning of chaperone activity, as well as in crosstalk within the proteostasis network. Research into this fascinating chaperone class has paved the way for new concepts of stress response to a changing cellular environment, and how this environment affects protein folding. However, most of these insights are derived from *in vitro* studies, and our knowledge of the native conformational changes and oligomerization states that chaperones utilize in cells is limited. Moreover, future work should address the issue of the substrate specificity of ATP-independent chaperones, and mechanism underlying the substrate release. We need to understand what drives the substrate specificity: sequence or structure, or both? Does the type of unfolding (e.g., oxidative, acidic, heat) affect successful substrate recognition? Is there cooperatively between different ATP-independent chaperones, and if so, does it depend on environmental conditions? Addressing these and related questions will not only furnish a deeper understanding of how the cell copes with environmental stresses but will provide great medical and biotechnological benefits.

### Conflict of interest statement

The authors declare that the research was conducted in the absence of any commercial or financial relationships that could be construed as a potential conflict of interest.
